# Inverse-Vulcanized Sulfur–Soybean Oil Polymers as Renewable Materials with Tunable Thermal Insulation Properties: Effect of Formulation and Biochar Incorporation

**DOI:** 10.3390/ijms27094044

**Published:** 2026-04-30

**Authors:** Luz M. Rovatta, Rodrigo E. de Prada, Acevedo Diego, Gustavo A. Monti

**Affiliations:** Departamento de Tecnología Química, Instituto de Investigaciones en Tecnologías Energéticas y Materiales Avanzados (IITEMA), Facultad de Ingeniería, Universidad Nacional de Río Cuarto (CONICET-UNRC), Río Cuarto 5800, Argentina; luzrovatta@ing.unrc.edu.ar (L.M.R.); rodrigodeprada@ing.unrc.edu.ar (R.E.d.P.)

**Keywords:** sulfur–soybean oil biopolymers, inverse vulcanization, biochar-reinforced composites, sustainable thermal insulation

## Abstract

Sulfur–soybean oil polymers with tunable thermal insulation properties were synthesized via inverse vulcanization of elemental sulfur and soybean oil and reinforced with biochar (BC) derived from spent barley biomass. Biopolymer films (F-BPs) with sulfur contents ranging from 20 to 80 wt% were prepared, and biochar-filled biocomposites (F-BP-Cs) were obtained using different filler loadings and processing routes. Their structural, morphological, thermal, mechanical, and surface properties were systematically analyzed to establish structure–property relationships, with particular focus on thermal transport behavior. Differential scanning calorimetry (DSC) revealed that sulfur contents ≤ 50 wt% favored the chemical incorporation of elemental sulfur into the polymer network via covalent bonding, significantly reducing the presence of free crystalline sulfur in the material. SEM images and porosity analysis revealed that BC incorporation and processing conditions significantly affected microstructural connectivity and air-filled porosity. As a result, F-BP-C materials exhibited low thermal conductivities, reaching values of ~0.033–0.039 W/(m·K), comparable to commercial insulating materials such as cork and polymeric foams. This reduction was attributed to increased structural disorder, high interfacial density, and enhanced phonon scattering within the heterogeneous polymer–BC–air system. These findings demonstrate the potential of these biocomposites as sustainable thermal insulating materials derived from industrial and agricultural waste.

## 1. Introduction

Polymeric materials play a central role in modern society due to their versatility, low density (~0.8–1.4 g·cm^−3^), ease of processing, and tunable physicochemical properties [1]. Conventional polymers, however, are predominantly derived from fossil resources, raising growing concerns related to sustainability, environmental persistence, and carbon footprint [2]. In response to these challenges, significant research efforts have been directed toward the development of bio-based and renewable polymeric materials that can partially or fully replace petroleum-derived counterparts while maintaining adequate performance for technological applications [3]. Bio-based materials have been developed to obtain properties similar to those of commercial polymers, particularly through strategic cross-linking and reinforcement. For instance, soy-based polyurethane adhesives reinforced with vanillin-derived Schiff base diols have shown tensile strengths exceeding 5000 kPa, matching high-performance industrial standards [4]). Similarly, the use of epoxidized soybean oil to synthesize polyurethane prepolymers allows for the creation of modified asphalt with thermal stability and resilience comparable to traditional petroleum-derived modifiers [5]. Furthermore, biochar-reinforced sulfur–soybean oil biocomposites have achieved thermal conductivities as low as ~0.033–0.039 W/(m·K), providing a sustainable insulation performance that directly competes with commercial materials such as polyethylene foam and cork [3]. Biopolymers, broadly defined as polymers derived from renewable biological resources, have emerged as promising alternatives for sustainable material design [6]; vegetable oils, polysaccharides, proteins, and other biomass-derived feedstocks have been extensively investigated due to their availability, low toxicity, and structural diversity [7]. Among them, vegetable oils are particularly attractive as polymer precursors because of their intrinsic unsaturation, which enables chemical modification and cross-linking reactions suitable for polymer synthesis [2] and film formation [8]. Soybean oil, in particular, is widely available, inexpensive, and rich in carbon–carbon double bonds, making it an ideal candidate for the development of bio-based polymeric networks [9]. Soybean oil is predominantly composed of unsaturated fatty acids, including approximately 18% monounsaturated and 68% polyunsaturated species, corresponding to a total unsaturation content of about 86% [10,11]. This high degree of unsaturation, also reflected in its iodine value (~126), provides a high density of carbon–carbon double bonds available for reaction during inverse vulcanization [2]). The versatility of soybean oil has enabled its successful application in various bio-based polymeric systems, effectively overcoming traditional mechanical limitations through innovative cross-linking strategies [4]. For instance, the incorporation of vanillin-derived Schiff base diols into soy-based polyurethane adhesives significantly enhances their tensile strength and thermal stability [4]. Furthermore, the synthesis of bio-based polyurethane prepolymers using epoxidized soybean oil has been shown to optimize the performance of modified asphalt, creating chemical networks that improve resilience across a wide range of temperatures [5]. Additionally, the selection of diverse diols and diisocynates in thermosetting soy-based polyurethane films allows for the fine-tuning of mechanical properties, establishing an optimal balance between flexibility and rigidity for high-performance applications [12].

In parallel, elemental sulfur has gained increasing attention as a sustainable feedstock for polymer synthesis. Sulfur is an abundant industrial byproduct of petroleum refining and natural gas processing, with annual production far exceeding its current demand. Traditional uses of sulfur are limited, prompting interest in valorization strategies that transform sulfur into functional materials [13]. The development of sulfur-rich polymers not only contributes to waste sulfur utilization but also enables access to materials with unique properties such as a high refractive index, chemical resistance, and thermal stability [14]. The importance of sulfur chemistry in polymer science has grown significantly due to the environmental and economic necessity of valorizing industrial waste [3,15]. Elemental sulfur is predominantly recovered as a by-product of crude oil refining through hydrodesulfurization and Claus processes, with global production reaching approximately 60–70 Mt·year^−1^ [13]. Inverse vulcanization has emerged as a key strategy to transform this low-cost residue into functional materials with unique properties, including high refractive indices, chemical resistance, and thermal stability [3,15,16]. Furthermore, sulfur-rich polymers synthesized through this green chemistry technique allow for the creation of durable, three-dimensional structures without the use of petroleum-based solvents or catalysts [2,3]. In fields such as agriculture, sulfur chemistry provides added value, as sulfur itself acts as an essential secondary plant nutrient and a fungicide [16,17]. When integrated into bio-based networks like those derived from soybean oil, sulfur chemistry enables the development of multifunctional materials for diverse applications, including energy storage, environmental remediation, and the controlled release of fertilizers [2,17,18].

A major breakthrough in sulfur polymer chemistry was the introduction of inverse vulcanization, a process in which molten elemental sulfur is stabilized through copolymerization with unsaturated organic comonomers [19,20]. Vegetable oils and their derivatives have proven to be particularly effective comonomers in inverse vulcanization reactions, leading to sulfur-based biopolymers that combine renewable carbon sources with surplus sulfur. These materials have been investigated for applications including cathode materials [21], optical components [22], environmental remediation [2], and coatings [14]. Beyond bulk polymer synthesis, the fabrication of sulfur-based films represents an important step toward practical applications, especially in surface-related technologies. Films produced from inverse vulcanized biopolymers offer advantages such as processability, flexibility, and the possibility of tailoring thickness and mechanical behavior [8]. However, sulfur-rich polymers often exhibit limitations in mechanical strength and dimensional stability, motivating the incorporation of reinforcing agents to enhance their performance [3]. In this context, biocomposites, materials composed of a polymer matrix reinforced with fillers derived from renewable resources, have attracted increasing interest [23]. The use of bio-based fillers not only improves mechanical and thermal properties but also reinforces the sustainability profile of the resulting materials [3]. Among these fillers, BC—a carbon-rich solid obtained from the pyrolysis or carbonization of biomass—has emerged as a multifunctional reinforcement material [24]. BC exhibits low density (~0.09–0.15 g·cm^−3^ [25]), high thermal stability, tunable porosity, and surface chemistry that can be adjusted through processing conditions such as carbonization temperature [26]. BC has been successfully incorporated into a wide range of polymer matrices, where it can act as a reinforcing filler, thermal barrier, and functional additive [27,28,29]. Its incorporation into polymer films has been shown to improve stiffness, thermal insulation performance, and dimensional stability, while potentially reducing material costs and environmental impact. Moreover, the use of BC derived from agricultural or agro-industrial residues contributes to circular economy strategies by valorizing waste biomass streams [30,31].

However, despite the growing interest in sulfur-based biopolymers, systematic studies correlating sulfur content, biochar incorporation, microstructure, and thermal transport mechanisms remain scarce. In particular, the role of interfacial phonon scattering and processing-induced porosity in governing thermal conductivity has not been comprehensively addressed. Elucidating these relationships is essential to enable the rational optimization of sulfur-rich biocomposites with enhanced thermal performance.

The reinforcement of sulfur-based F-BP-C represents a particularly promising approach for the development of sustainable thermal insulating materials [3]. Thermal insulation materials are critical in energy-efficient buildings, packaging, and industrial systems, yet many commercial solutions rely on non-renewable or environmentally problematic materials [32]. Sulfur-based biopolymers, combined with BC fillers, offer a unique combination of low thermal conductivity (~0.03–0.10 W/(m·K) [3,33,34]), chemical resistance, and renewable content, positioning them as attractive candidates for next-generation insulating films and coatings [35]. In this work, we report the synthesis of inverse-vulcanized biopolymers based on elemental sulfur and soybean oil with varying sulfur content, their processing into flexible bio-based films, and the development of novel F-BP-C derived from carbonized spent barley biomass. The influence of sulfur content and BC characteristics on the physicochemical and mechanical properties of the resulting materials is systematically investigated, with particular emphasis on structure–property relationships and their implications for thermal insulation and surface-related applications.

## 2. Results and Discussion

### 2.1. Characterization of Materials

#### 2.1.1. Visual Appearance of the Materials

**Figure 1:**
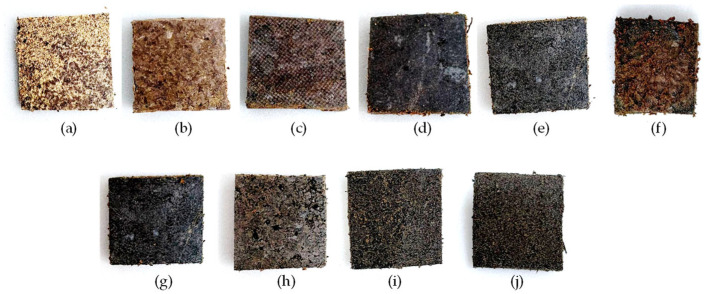
Representative photographs of the synthesized materials: (**a**) F-BP80, (**b**) F-BP70, (**c**) F-BP60, (**d**) F-BP50, (**e**) F-BP40, and (**f**) F-BP30 (**top row**); (**g**) F-BP60-C15, (**h**) F-BP60-C25, (**i**) F-BP40-C15, and (**j**) F-BP40-C25 (**bottom row**).

Figure 1 presents representative photographs of the synthesized materials. The first row corresponds to F-BP films with decreasing sulfur content (80–30 wt%). A clear transition in color and surface texture is observed: high-sulfur samples (F-BP80–F-BP70) show lighter, heterogeneous, and granular surfaces, consistent with sulfur-rich domains, while lower sulfur contents (F-BP60–F-BP30) lead to darker, more uniform and compact films.

The second row shows F-BP-Cs with different sulfur contents (40 and 60 wt%) and BC loadings (15 and 25 wt%). The incorporation of BC produces darker materials with increased surface roughness and heterogeneity. Higher BC content (25 wt%) enhances these features, leading to more pronounced texturing, which reflects the dispersion of carbonaceous particles within the polymer matrix.

#### 2.1.2. FT-IR

**Figure 2:**
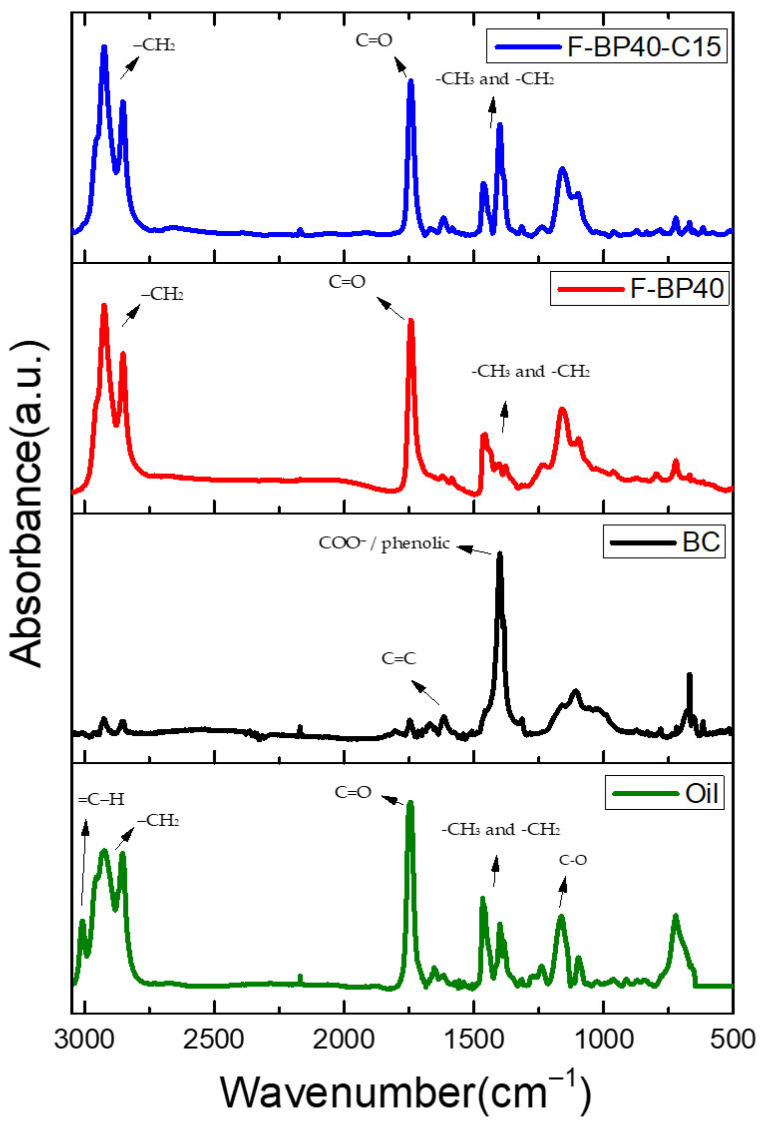
FT-IR spectra of soybean oil (green line), BC obtained from barley biomass at 200 °C (black line BC), F-BP40 (biopolymer composed of sulfur and soybean oil (red line), and F-BP40-C15 composite biopolymer incorporating BC (blue line).

The chemical composition of the materials was analyzed by FT-IR. Elemental sulfur exhibits very limited infrared activity, showing only weak absorptions below 500 cm^−1^ associated with S_8_ ring vibrations [36,37]. Therefore, the analysis focuses primarily on the structural evolution of soybean oil and its derived polymers. Figure 2 shows the FT-IR spectra of soybean oil (Oil), BC obtained from barley waste carbonized at 200 °C (BC-200), a sulfur–soybean oil copolymer (F-BP40), and a composite biopolymer containing dispersed BC (F-BP40-C15). These spectra provide valuable insight into the structural evolution of materials during synthesis and modification. Soybean oil (Figure 2, green line) exhibits well-defined peaks corresponding to its triglyceride structure. The bands observed near 3010 cm^−1^ correspond to the =C–H stretching of *cis* double bonds, while the intense peaks at 2922 cm^−1^ and 2853 cm^−1^ are associated with asymmetric and symmetric –CH_2_ stretching vibrations, respectively. A strong absorption around 1745 cm^−1^ is indicative of carbonyl groups (C=O), and bands in the 1164 cm^−1^ region arise from C-O stretching. These features are consistent with previous reports on vegetable oils used as renewable precursors for polymeric materials [38]. BC (Figure 2, black line) obtained from barley waste at 200 °C reveals a broader and less intense spectrum, reflective of partial pyrolysis. Notably, the broad band near 3400 cm^−1^ suggests residual hydroxyl groups (-OH), likely originating from lignocellulosic components. Peaks around 1600 cm^−1^ may be attributed to aromatic C=C stretching, while the intense band near 1404 cm^−1^ likely corresponds to carboxylate symmetric stretching or phenolic group vibrations, reflecting the presence of oxygenated functionalities in the BC structure [39,40]. The relatively low carbonization temperature preserves oxygenated functional groups, which are important for further interaction with polymer matrices.

The spectrum of the F-BP40 (Figure 2, red line) formed via inverse vulcanization of sulfur and soybean oil reveals significant structural transformation. The reduction of =C-H stretching signals (~3010 cm^−1^) indicates successful consumption of unsaturated bonds. Furthermore, significant changes can be observed around 1400 cm^−1^ in the spectrum of the BP compared to Oil. These spectral modifications suggest alterations of the -CH_3_ and -CH_2_ in the oil, likely due to their involvement in the formation of new sulfur-containing bonds. The changes in band intensity and position reflect structural and chemical transformations consistent with sulfur incorporation and the formation of a cross-linked polymer network. These results are consistent with those previously reported by Farioli et al. [2]. The authors attributed the disappearance of the unsaturated bands to the reaction of the double bonds with sulfur to cross-link the oil molecules through sulfur–sulfur bonds. Therefore, these findings support the success of inverse vulcanization. The spectral evidence confirms that sulfur radicals reacted effectively with alkene functional groups in the oil, leading to the formation of a thermosetting polymer with a chemically modified structure.

The composite biopolymer F-BP40-C15 (Figure 2, blue line) containing BC maintains the primary spectral features of F-BP40 but exhibits notable broadening and slight shifts in several regions, especially between 1500 and 1000 cm^−1^. Notably, the decrease in intensity of the 1400 cm^−1^ band suggests either dilution of aliphatic functionalities or specific interactions between the polymer matrix and the carbon surface. These changes support the effective integration of carbonaceous components into the polymer structure. Additionally, these modifications are indicative of physical and potentially chemical interactions between the BC surface and the polymer matrix. Similar effects have been observed in studies where BC was incorporated into synthetic or bio-based polymers, often leading to improved thermal, mechanical, and barrier properties [41]. Overall, the FT-IR analysis confirms the structural transitions involved in the synthesis of sulfur–oil-based polymers and highlights the role of BC as a functional filler capable of interacting with the matrix.

#### 2.1.3. SEM Images

**Figure 3:**
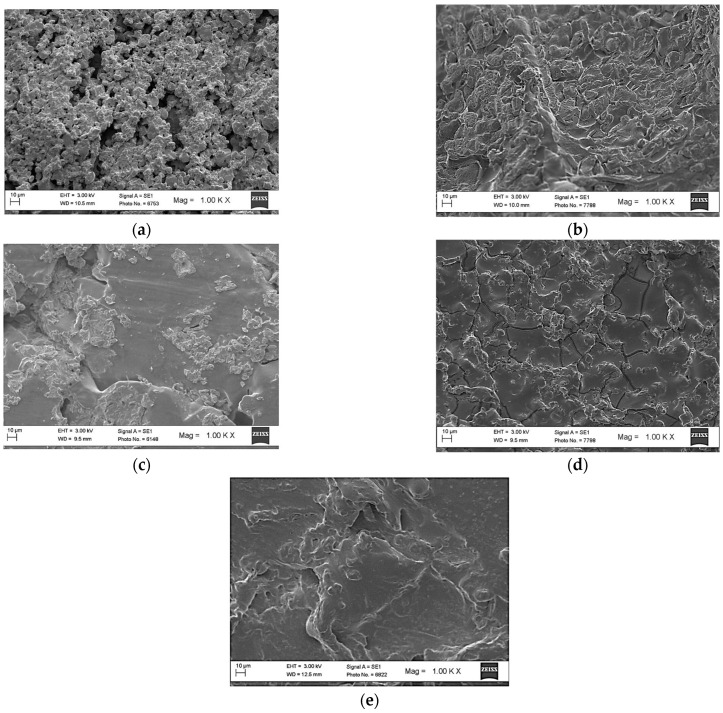
SEM images for biopolymer formulations with different weight percentages of sulfur: (**a**) F-BP70, (**b**) F-BP60, (**c**) F-BP50, (**d**) F-BP40, and (**e**) F-BP30.

To investigate the surface morphology of the biopolymer formulations, SEM imaging was performed. Figure 3 shows SEM micrographs of sulfur–soybean oil biopolymers prepared with varying sulfur content (F-BP70, F-BP60, F-BP50, F-BP40, F-BP30). It can be seen that as the sulfur content increases, noticeable differences in the surface morphology are observed. F-BP70 exhibits a rough, cracked surface with irregular, fragmented domains, indicating phase separation and brittleness. This phase separation probably is due to the elemental sulfur remaining in the polymer. This morphology is characteristic of sulfur-rich formulations, where excess elemental sulfur crystallizes or segregates during cooling. This type of morphology, associated with sulfur microcrystals embedded within the polymer, is consistent with observations reported by Salman et al. [42]. Similarly, the F-BP60 sample maintains a heterogeneous and porous texture, suggesting that it is partially compatible with the organic phase but still dominated by sulfur continuity. As the sulfur content decreases (F-BP50 and F-BP40), the morphology becomes more uniform, showing a continuous matrix without large cracks or heterogeneous domains visible. However, the uniform and smoother texture observed in the SEM images do not exclude the presence of micro-holes or internal pores. Moreover, F-BP50 shows a smoother surface with fewer cracks, suggesting improved miscibility between the polymeric sulfur network and soybean oil; this behavior could be due to almost all the sulfur being used during the synthesis, leading to a small amount of free sulfur in the biopolymer. The F-BP40 material shows a continuous matrix with well-distributed features and minimal phase segregation, indicating optimal cross-linking and compatibility at intermediate sulfur concentrations. At the lowest sulfur content (in F-BP-30), the microstructure appears more amorphous and featureless, with a relatively flat surface and minimal textural variation. This suggests a dominant organic phase with limited sulfur domains, resulting in a softer and more homogeneous material. The reduced presence of crystalline sulfur domains is consistent with the lower inorganic content during the synthesis.

**Figure 4:**
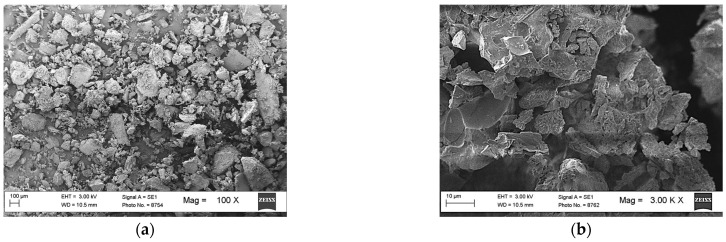
SEM images of biomass-derived BC at (**a**) low (100×) and (**b**) high magnification (3000×).

SEM micrographs of the BC filler at different magnifications are shown in Figure 4. At low magnification (Figure 4a), the BC exhibits a highly heterogeneous particle size distribution, with particle sizes consistent with the sieved fraction (<180 µm) and predominantly irregular, angular morphologies characteristic of biomass-derived carbon materials [43]. The absence of smooth or spherical particles indicates a brittle fragmentation mechanism, resulting in rough and highly fragmented surfaces with poorly defined particle boundaries. Such disordered morphology is typically associated with BC produced at moderate carbonization temperatures [44], where partial retention of the original biomass structure leads to an underdeveloped carbon framework with increased surface roughness and porosity [45]. While this morphology provides a large interfacial area and surface heterogeneities, it may also limit the intrinsic mechanical integrity of the carbon filler. Higher-magnification images (Figure 4b) reveal a markedly rough surface with the presence of micro- and mesoporous features, as well as sharp edges and surface cavities. This porous and irregular morphology significantly increases the effective interfacial area between the BC particles and the polymer matrix, facilitating enhanced physical interaction and mechanical interlocking between phases [46]. Such features are expected to influence sulfur mobility during inverse vulcanization, potentially acting as preferential sites for sulfur confinement or heterogeneous nucleation during cooling.

**Figure 5:**
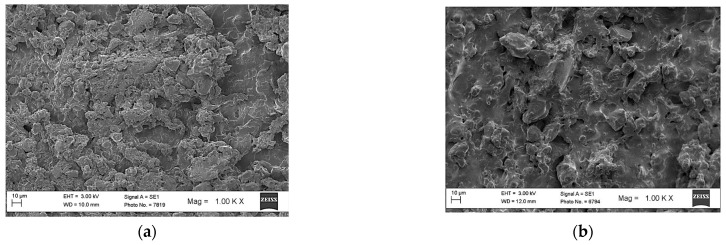

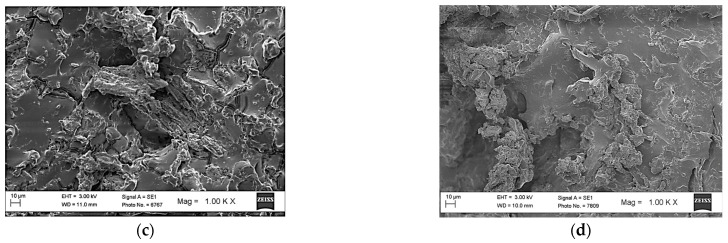
SEM images for (**a**) F-BP60-C15, (**b**) F-BP60-C25, (**c**) F-BP40-C15, and (**d**) F-BP40-C25.

Figure 5 compares SEM micrographs of F-BP-C obtained with a fixed sulfur-oil ratio and two different concentrations of BC (15 wt% and 25 wt%). As can be noted, the presence of BC significantly alters the morphology of the polymer matrix. For both the F-BP40 and F-BP60, the incorporation of 15% BC leads to the appearance of discrete dark regions and embedded aggregates within the polymeric matrix. These regions possibly correspond to BC particles partially embedded in the organic sulfur network. The F-BP60-C15 (Figure 5a) shows evident carbon-rich domains that could serve as reinforcement to the F-BP-C. In the same sense, when the BC content increases to 25% (Figure 5b) a more pronounced dispersion of BC structures is observed. In both F-BP40-C25 and F-BP60-C25, the BC is more uniformly distributed and integrated into the polymer matrix, with a slight increase in surface roughness and porosity compared with F-BP-C25. The higher BC content increases surface heterogeneity but may also improve mechanical stability. In agreement with previous studies, the development of interconnected BC domains within polymer matrices has been reported to improve stress transfer efficiency and load-bearing capacity, while potentially limiting ductility due to restricted polymer chain mobility [3,29,47]. The morphological characteristics observed here are consistent with such reinforcing mechanisms.

Overall, SEM analysis confirms that sulfur content strongly affects the morphology of F-BP, as higher sulfur content results in more brittle and fractured textures, while intermediate ratios improve a more homogeneous surface. On the other hand, the incorporation of BC to obtain the biocomposites introduces particulate characteristics and increases structural complexity, allowing the tuning of the material properties that could be exploited in different specific applications such as adsorption, reinforcement, and insulation.

#### 2.1.4. Thermal Properties

**Figure 6:**
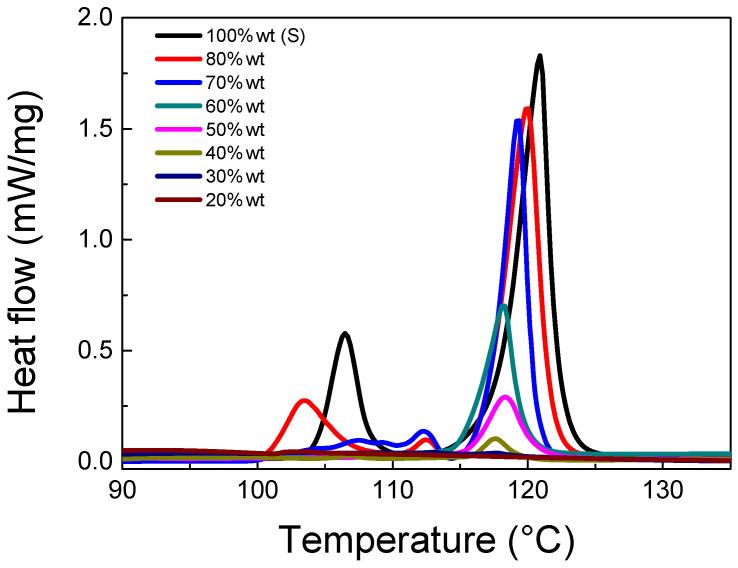
DSC thermograms of pristine sulfur, biopolymer formulations with varying sulfur contents: 20 wt% (BP20, brown line), 30 wt% (BP30, dark blue line), 40 wt% (BP40, olive line), 50 wt% (BP50, pink line), 60 wt% (BP60, green line), 70 wt% (BP70, blue line), 80 wt% (BP80, red line) and 100% (S, black line).

To investigate the thermal behavior of the BP, DSC thermograms of elemental sulfur and sulfur–soybean oil biomaterials with varying sulfur contents (80–20 wt%) and pristine sulfur are shown in Figure 6. Also, the DSC was employed to quantify the residual free sulfur (S_8_) in BP by integrating the endothermic peak corresponding to the melting of crystalline sulfur (~119 °C). Table 1 summarizes the DSC peak areas and the calculated free sulfur percentages for each formulation [19,48].

Pristine sulfur (Figure 6, black line) displays two distinct thermal events: a minor endothermic transition at 107 °C, corresponding to the orthorhombic-to-monoclinic allotrope transformation (λ-transition), and a sharp endothermic peak at 119 °C, attributed to the melting of crystalline S_8_ rings. Similar behavior has been reported by He et al. [49], and Ficara et al. [50]; the high intensity and definition of these peaks confirm the high crystallinity of elemental sulfur.

In BP with high sulfur content (80 wt%, 70 wt%, 60 wt%), a melting peak around 119 °C is still observed, although with reduced intensity. This result indicates the presence of residual unreacted sulfur that was not fully incorporated into the biopolymeric network during inverse vulcanization reaction [2]. The progressive decrease in peak intensity with decreasing sulfur content indicates the gradual consumption of elemental sulfur and a lower fraction of free crystalline sulfur in the BP materials. However, at intermediate sulfur contents (e.g., BP40), a deviation from this proportional trend in heat flow is observed. This behavior can be attributed to the partial confinement of sulfur within the polymer matrix and its reduced ability to organize into well-defined crystalline domains, leading to decreased crystallinity and lower melting enthalpy. This is consistent with the formation of a more homogeneous, amorphous polysulfide network; in sulfur chains, the sulfur used in the reaction mixture became covalently cross-linked with the unsaturated sites of soybean oil triglycerides [19,48,49,50,51]. In addition, within the investigated temperature range (25–150 °C), no glass transition or other thermal events were detected for these low-sulfur formulations, further supporting the formation of thermally uniform and highly cross-linked polymer networks under the conditions studied.

A clear trend is observed as the sulfur content in the initial formulation increases: the proportion of unreacted sulfur in the final material rises significantly. Formulations with ≤30 wt% sulfur exhibit minimal free sulfur (<0.4%), indicating nearly complete incorporation of sulfur into the polymeric network. In contrast, formulations with higher sulfur content show progressively larger melting endotherms; for the reaction mixture using 80 wt% of sulfur, the final material contains over 66% free sulfur. Previous studies have reported that the use of higher initial sulfur ratios leads to a higher amount of unreacted sulfur in the resulting polymers. Ghumman et al. [52] reported that inverse-vulcanized copolymers of palm oil and sulfur showed increasing amounts of unreacted sulfur with higher sulfur feed ratios, as evidenced by DSC and XRD analyses. Also, studies with synthetic dienes indicate the presence of unreacted sulfur in formulations with higher sulfur loads by not suppressing its crystallization [14].

The presence of unreacted sulfur is critical, as it can lead to issues such as sulfur blooming, reduced mechanical stability, and potential leaching in applications. Therefore, optimizing the sulfur-to-oil ratio is essential to minimize free sulfur content and enhance the material’s performance and stability.

It has been reported that the presence of free sulfur in polymers obtained by inverse vulcanization can compromise structural homogeneity and may also negatively affect the mechanical and thermal stability of the material [53]. The absence of free crystalline sulfur in these lower-S formulations is advantageous, as it prevents issues such as sulfur bloom, crystallization over time, and mechanical instability. These findings suggest that sulfur contents ≤ 50 wt% result in better thermal integration and more stable polymeric structures, making them more suitable for long-term applications where phase separation or sulfur leaching must be avoided.

**Figure 7:**
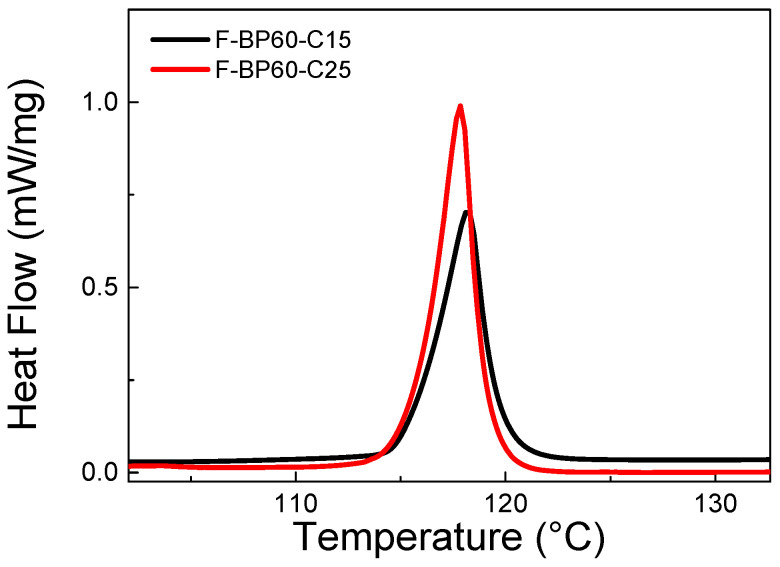
DSC thermograms of sulfur–soybean oil F-BP-C containing 60 wt% sulfur with different BC contents: F-BP60-C15 (black line) and F-BP60-C25 (red line).

Figure 7 compares the DSC thermograms of sulfur–soybean oil F-BP containing 60 wt% sulfur and different BC contents (15 and 25 wt%). Both formulations exhibit an endothermic peak at ~119 °C, associated with the melting of residual crystalline sulfur (S_8_), comparable to that observed for BP60. This indicates that a measurable fraction of sulfur remains unincorporated into the polymer network even in the presence of carbonaceous fillers.

Interestingly, the F-BP60-C25 (Figure 7, red line) sample displays a sharper and more intense melting peak compared to F-BP60-C15. This suggests that the presence of BC in the reaction mixture may hinder sulfur incorporation into the polymer network, potentially due to steric effects or interfacial incompatibility between the sulfur phase and the carbonaceous filler. Alternatively, BC particles could act as thermal insulators or nucleation sites, promoting the crystallization of unreacted sulfur domains during cooling [54]. The slight shift and narrowing of the melting peak in F-BP60-C25 may also indicate changes in the crystalline morphology of sulfur induced by the filler. These observations suggest that while BC may confer additional properties (e.g., mechanical reinforcement or adsorption capacity) [55], it could also affect sulfur dispersion and network formation during the polymerization process. This is consistent with the highly irregular and porous morphology of the BC filler (Figure 4), which provides a large interfacial area and surface heterogeneities that may locally influence sulfur diffusion and cross-linking during inverse vulcanization.

#### 2.1.5. Contact Angle

**Figure 8:**
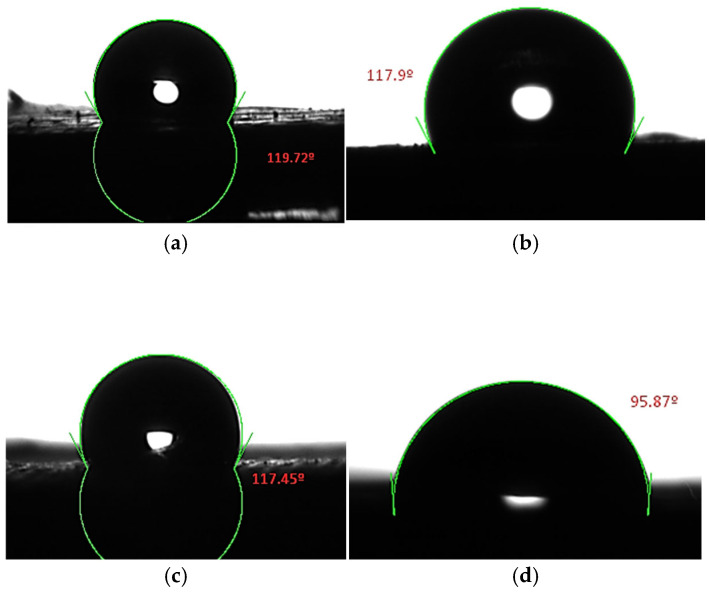
Water CA measurements of biopolymers with different sulfur contents: (**a**) F-BP70, (**b**) F-BP60, (**c**) F-BP50, and (**d**) F-BP30.

Surface wettability was assessed via static CA measurements, as shown in Figure 8. The F-BPs with 70 wt%, 60 wt%, and 50 wt% sulfur exhibit high contact angles of 119.72°, 119.90°, and 117.45°, respectively. These results indicate a predominantly hydrophobic surface. In contrast, the sample with 30 wt% sulfur shows a significantly reduced CA of 95.87°, denoting a drastic increase in surface wettability and polarity. The high CA values of F-BP70, F-BP60, and F-BP50 can be attributed to the dominance of sulfur in the polymer matrix, resulting in surfaces that are chemically less polar and more hydrophobic. Elemental sulfur and sulfur-rich domains have poor affinity for water, which is consistent with previous reports on inverse vulcanized polymers [56]. The slight decrease from 119.72° to 117.45° between F-BP70 and F-BP50 suggests that up to 50 wt% oil incorporation does not significantly disrupt the hydrophobicity of the sulfur-rich matrix.

However, the drastic decrease in CA to 95.87° of the F-BP30 sulfur shows a substantial modification of the chemical surface. This change could reflect an increased presence of polar functionalities introduced by soybean oil, which are known to enhance interaction with water via hydrogen bonds and reduce hydrophobicity, as has been observed in the context of other polymeric materials with ester and hydroxyl groups [47]. Several authors have probed the finding that the smoother surface morphology (as shown in the F-BP30 SEM images) produces a more hydrophilic surface. Genzer et al. [57] report that the maximum contact angle of a water droplet on a smooth, non-textured surface is approximately 130°. However, surfaces with micro- or nanoscale texturing, such as the F-BP70 and F-BP50, can exhibit significantly higher contact angles. On these biopolymer surfaces, the water droplet clearly adopts the Cassie–Baxter state to minimize its overall free energy, and the liquid does not fully penetrate the surface texture, leaving air pockets trapped beneath the droplet [58]. On the other hand, the F-B30 presents a low contact angle value that can be attributed not only to the more hydrophilic chemical composition of the surface but also to the more uniform, smoother, and less rough surface, as the SEM images show (Figure 3e).

These findings confirm that surface wettability in sulfur–oil BP can be modulated through compositional tuning. Materials with higher sulfur content retain hydrophobic characteristics, suitable for applications such as water-repellent coatings or encapsulation, while lower sulfur content enhances surface polarity, potentially benefiting sorption or adhesion applications.

**Figure 9:**
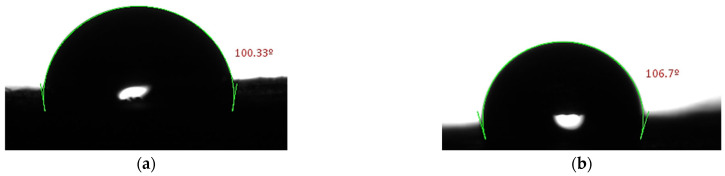
Water CA measurements of biocomposites (**a**) F-BP60-25 HC and (**b**) F-BP60-25 CC.

To further elucidate the effect of carbon incorporation, CA measurements were performed on biocomposites containing the same sulfur content (60 wt%) and 25 wt% biochar. The results are presented in Figure 9. Compared with pristine F-BP60 (119.90°), the F-BP60-25 composite (Figure 9a) exhibits a lower CA (110.33°), indicating a moderate increase in surface wettability upon BC incorporation. Although BC is mainly carbonaceous, its production at relatively low carbonization temperature (200 °C) preserves oxygen-containing functional groups, as suggested by FT-IR analysis. Partial exposure of these polar fractions on the composite surface increases the surface free energy and enhances hydrogen bond interactions with water, leading to a decrease in contact angle. Additionally, SEM images reveal increased surface heterogeneity after BC incorporation (Figure 5). In a sulfur-rich matrix that is intrinsically hydrophobic, the introduction of dispersed carbon domains modifies the local surface chemistry and roughness, potentially promoting partial liquid penetration into surface irregularities. This combined chemical and morphological effect accounts for the consistent reduction in CA relative to pristine F-BP60, while maintaining an overall hydrophobic character (CA > 100°).

On the other hand, in previous work, we investigated the structural and mechanical properties of films made from these BPs to establish structure–property relationships. These materials exhibited relatively low bulk densities (1.03–1.43 g/cm^3^) and porosity (2.30–5.71%) [3,59]. Also, a porous internal morphology was found, especially in formulations with BC, where the filler acted not only as a reinforcing phase but also as a microstructural modifier, promoting greater free volume and interfacial heterogeneity. Mechanical tests revealed that increasing sulfur content significantly enhanced stiffness and tensile strength, attributed to the formation of denser S–S cross-linked networks. Furthermore, the incorporation of BC significantly enhanced stiffness and strength while preserving adequate flexibility of the polymeric network, indicating the formation of mechanically robust yet lightweight composite structures.

These effects were attributed to improved stress transfer at the polymer–BC interface and to the constrained mobility of sulfur-rich polymer chains within the porous framework [46,60]. In addition, in a preliminarily evaluation of the thermal insulation potential using infrared thermographic, it was observed that all formulations maintained lower surface temperatures than the control, suggesting effective thermal resistance.

### 2.2. Thermal Conductivity

#### Effect of Sulfur Content and Porosity

The effect of sulfur content and BC filler on the thermal conductivity of the synthesized materials was systematically evaluated, and the results are summarized in Table 2. A clear tendency is observed in which thermal conductivity increases as sulfur content decreases. For instance, F-BP70 shows a thermal conductivity of 0.0404 W/(m·K) compared to 0.0610 W/(m·K) for F-BP30. This is a significant reduction when contrasted with pure elemental sulfur (λ_t_ ≈ 0.205 W/(m·K) [61]), indicating that the chemical structure resulting from inverse vulcanization plays a key role in suppressing heat transport. This reduction in λ_t_ can be primarily attributed to enhanced phonon scattering induced by the chemically heterogeneous and amorphous polymeric architecture. In polymers systems, heat is mainly conducted through phonons—quantized lattice vibrations—and the presence of structural disorder significantly limits their transport [62]. The incorporation of long-chain organic structures from soybean oil, along with the formation of an irregular sulfur-based network via inverse vulcanization, introduces numerous phonon-scattering centers such as chemical cross-links, chain irregularities, and interfacial defects. These features hinder the free propagation of phonons, thereby reducing the material thermal conductivity. In amorphous and polymeric systems such as the ones studied here, phonon mean free paths are strongly suppressed by molecular disorder and heterogeneous interfaces, which plays a key role in lowering thermal transport efficiency [63,64].

In addition to chemical disorder, porosity plays a crucial role in further suppressing heat transfer. Highly porous formulations, such as F-BP70 (φ = 5.71%) and F-BP60-C25 (φ = 5.26%), display some of the lowest λ_t_ values in the series (0.0404 and 0.0393 W/(m·K), respectively). This behavior could be attributed to the combined effect of the reduction of solid pathways and the entrapment of air (0.026 W/(m·K) [65]) within the matrix. These results align with prior reports on sulfur-based porous materials synthesized via inverse vulcanization, where increased porosity led to suppressed heat transport due to diminished connectivity in the solid framework [39].

The combined influence of polymer matrix, BC filler, and air-filled pores can be rationalized within the framework of effective medium theory. In an approximation, the Maxwell–Eucken model describes the effective thermal conductivity ($\lambda_{eff}$) of a continuous polymeric phase containing dispersed low-conductivity inclusions according to [66,67,68]:

(1) $$\lambda_{eff}=\lambda_{m}\frac{\lambda_{d}+2\lambda_{m}-2\phi \left(\lambda_{m}-\lambda_{d}\right)}{\lambda_{d}+2\lambda_{m}+\phi \left(\lambda_{m}-\lambda_{d}\right)}$$

where $\lambda_{m}$ is the thermal conductivity of the polymer matrix, $\lambda_{d}$ corresponds to the dispersed phase (air or BC), and ϕ is the volume fraction of inclusions. This model predicts a monotonic decrease in $\lambda_{eff}$ with increasing pore volume fraction.

**Figure 10:**
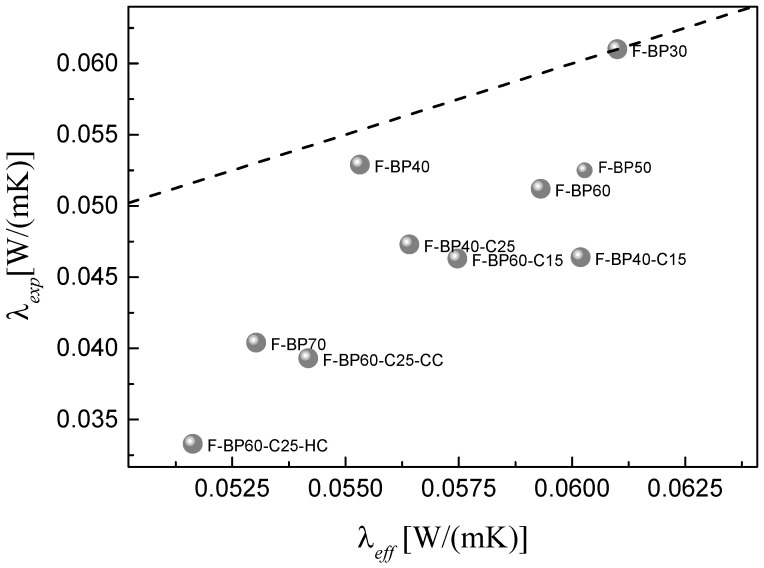
Comparison between theoretical thermal conductivity predicted by the Maxwell–Eucken model ($\lambda_{eff}$), and experimentally measured values ($\lambda_{exp}$) predict the effective thermal conductivityfor all formulations. The dashed line represents λ*_exp_* = λ*_eff_*.

To further assess the contribution of porosity to thermal transport, the intrinsic thermal conductivity of the solid phase ($\lambda_{m}$) was estimated from the least porous formulation (F-BP30, φ = 2.30%), yielding $\lambda_{m}$ ≈ 0.064 W/(m·K). This value was subsequently used to predict the $\lambda_{eff}$ of the remaining samples using the Maxwell–Eucken model considering air-filled pores as the dispersed phase. As shown in Figure 10, the model correctly captures the decreasing trend of thermal conductivity with increasing porosity. However, the experimentally measured values are systematically lower than the theoretical predictions. For the most porous formulation, F-BP60-C25-HC (φ = 6.15%), the model predicts $\lambda_{eff}$ ≈ 0.052 W/(m·K), whereas the experimental value is 0.033 W/(m·K), corresponding to an additional reduction of ~35%. This deviation indicates that volumetric porosity alone cannot fully account for the strong suppression of heat transport.

Given that the thermal conductivity of air is more than one order of magnitude lower than that of the sulfur-based polymer matrix, the Maxwell–Eucken expression becomes strongly governed by the pore volume fraction ϕ. Under such high conductivity contrast ($\lambda_{d}$≪$\lambda_{m}$), even moderate increases in porosity lead to a pronounced decrease in $\lambda_{eff}$, as experimentally observed for the highly porous formulations (F-BP70 and F-BP60-C25) [3]. This highlights that φ acts as a primary parameter governing thermal transport in these composites. However, due to the simultaneous presence of polymer, BC, and air, a more realistic description is given by the effective medium approximation [69], which considers the contribution of all phases to heat transport. Within this multiphase framework, the effective thermal conductivity arises from the balance between the relatively low thermal conductivity of the BC, the polymeric matrix, and the air-filled porosity.

Beyond volumetric effects, the large number of interfaces present in these heterogeneous systems plays a decisive role in suppressing heat transport. Phonon scattering at the polymer–BC, polymer–air, and BC–sulfur interfaces generates significant interfacial thermal resistance (Kapitza resistance [70]), further reducing λ_t_. The discordance of vibrational spectra between sulfur-rich polymer chains, carbonaceous BC domains, and the gaseous phase leads to inefficient phonon transmission across interfaces, thereby enhancing thermal insulation. Consequently, thermal transport in these composites is governed by a synergistic interplay between volumetric dilution effects and multiscale interfacial phonon scattering, which together amplify heat-flow suppression beyond effective medium predictions.

Given the strong dependence of thermal transport on porosity and microstructural connectivity, the influence of processing conditions on the physical, thermal, and mechanical properties was further evaluated for the F-BP60-C25. The cold-pressed material exhibits a lower bulk density (1.2093 g/cm^3^) and a higher porosity (φ = 6.15%) compared to the hot-pressed counterpart (density = 1.3259 g/cm^3^, φ = 5.26% [3]), resulting in a further reduction of thermal conductivity. In particular, the thermal conductivity of the cold-pressed F-BP60-C25 sample (λ_t_ = 0.0333 W/(m·K)) is approximately 15% lower than that of the hot-pressed material (λ_t_ = 0.0393 W/(m·K), consistent with the increased air volume fraction within the polymeric matrix. These differences in density and porosity can be directly attributed to the distinct reactive compression regimes imposed during processing. In inverse-vulcanized sulfur polymers, higher temperature and pressure during hot pressing favor interfacial S–S metathesis and effective interparticle fusion, leading to enhanced densification and reduced residual porosity. In contrast, cold pressing limits chain mobility and suppresses interfacial reactive rearrangements, resulting in incomplete consolidation and the retention of a larger population of air-filled voids within the bulk material. This processing–structure relationship is consistent with previous reports on sulfur-based polymer networks processed by reactive compression molding, where the pressure–temperature window controls the extent of particle fusion and void elimination [8]. Consequently, the increased porosity observed in the cold-pressed F-BP60-C25 sample amplifies phonon scattering and disrupts continuous heat-transfer pathways. This behavior is in good agreement with effective medium models, in which an increase in pore volume fraction leads to a monotonic decrease in effective thermal conductivity due to the progressive replacement of solid heat-transfer pathways by air-filled voids. Given the large thermal conductivity contrast between the sulfur-based polymer matrix and air, even moderate increases in porosity result in a noticeable suppression of heat transport.

Therefore, the exceptionally low thermal conductivity values achieved in highly porous formulations (particularly in samples such as F-BP60-C25) result from a synergistic combination of a high air volume fraction, the low intrinsic thermal conductivity of the BC phase, and enhanced interfacial phonon scattering.

**Figure 11:**
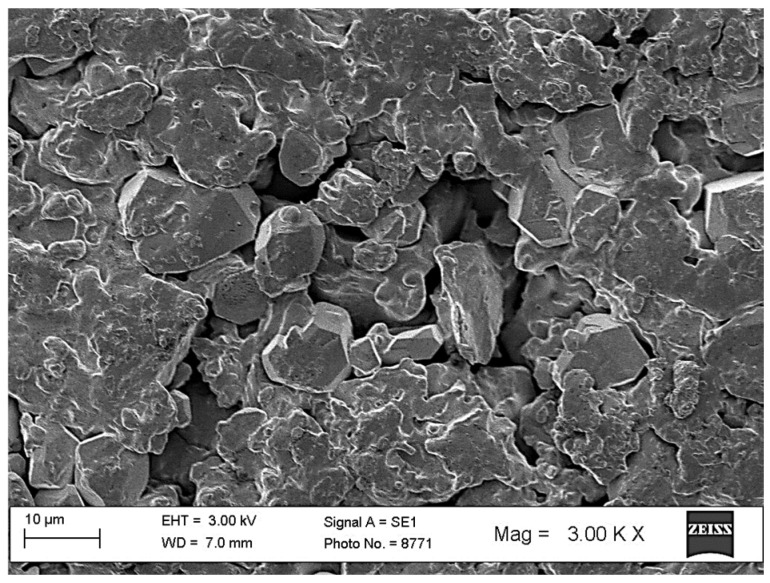
SEM micrographs of the surface of F-BP60-C25 films processed by cold pressing.

This highlights that thermal insulation in these sulfur-based F-BP materials is governed not only by porosity but also by interfacial thermal resistance arising from heterogeneities spanning multiple structural length scales. These microstructural effects are further supported by SEM micrographs of the film surfaces, which reveal marked differences in surface topography and phase continuity induced by the processing method. The hot-pressed samples (Figure 5b) exhibit a comparatively smoother and more compact surface, characterized by improved interparticle contact between the sulfur-based polymer matrix and the BC domains, as well as reduced occurrence of surface voids and microcracks. In contrast, the cold-pressed films (Figure 11) display a rougher surface morphology with a higher density of surface pores, interfacial gaps, and poorly fused domains, indicative of limited polymer flow and incomplete consolidation during processing.

CA values for F-BP60-25-HC and F-BP60-25-CC were measured (Figure 9a,b) to evaluate the processing conditions. The hot-pressed sample exhibits a slightly higher CA (Figure 9a, 100.33°) than the cold-pressed (Figure 9b, 106.70°). Since both samples share identical chemical composition, this difference is attributed primarily to processing-induced variations in surface microstructure rather than to changes in surface chemistry. SEM analysis (Figure 5b) indicates that hot pressing enhances polymer chain mobility and interparticle fusion, promoting improved consolidation and partial surface reflow. The applied thermal treatment may also facilitate further rearrangement within the sulfur network, resulting in a more homogeneous and smoother surface. In contrast, cold pressing limits molecular mobility and interfacial reorganization, preserving surface irregularities and microstructural heterogeneity (Figure 11). Because the composite remains intrinsically hydrophobic, the observed behavior can be interpreted within the Wenzel model [71], where surface roughness amplifies the intrinsic wettability of the material. Accordingly, the higher apparent CA of the cold-pressed sample is consistent with roughness-induced enhancement of hydrophobicity, whereas the smoother surface obtained by hot pressing reduces this amplification effect. Although both materials remain hydrophobic, these findings demonstrate that wettability is governed not only by composition but also by processing-driven surface organization.

**Figure 12:**
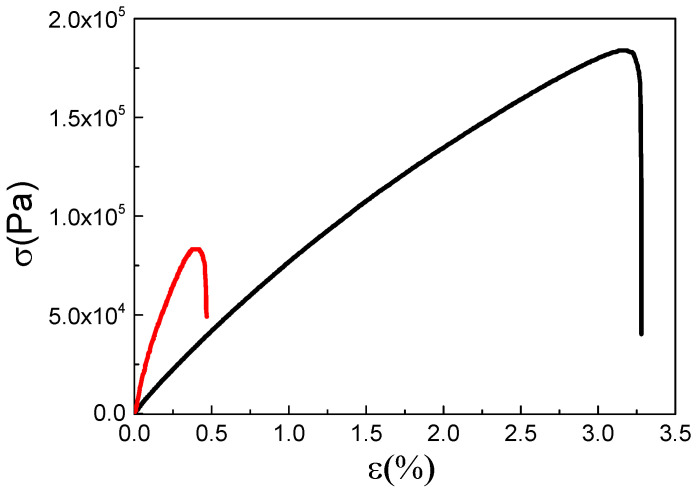
Stress–strain curves of F-BP60-C25 composites processed by hot pressing (black line) and cold pressing (red line).

On the other hand, the higher porosity of the cold-pressed material severely compromises its mechanical integrity. Tensile tests (Figure 12) show that although the cold-pressed sample exhibits a higher apparent Young modulus (19.15 MPa) than the hot-pressed material (5.83 MPa), it displays a significantly lower tensile strength (0.088 MPa vs. 0.185 MPa) and reduced elongation at break (Table 3). This combination indicates a brittle mechanical response, characterized by early failure and limited load-bearing capacity, rendering the cold-pressed films unsuitable for self-supporting applications. In contrast, hot pressing yields mechanically robust films while maintaining a low thermal conductivity within the range of efficient insulating materials. The improved mechanical performance of the hot-pressed samples is attributed to enhanced material consolidation and surface continuity, which promote more effective stress transfer and structural cohesion, despite a lower apparent Young modulus. Therefore, hot pressing emerges as the most suitable processing route for the fabrication of sulfur–soybean oil F-BP, offering an optimal balance between mechanical integrity and thermal insulation performance.

**Figure 13:**
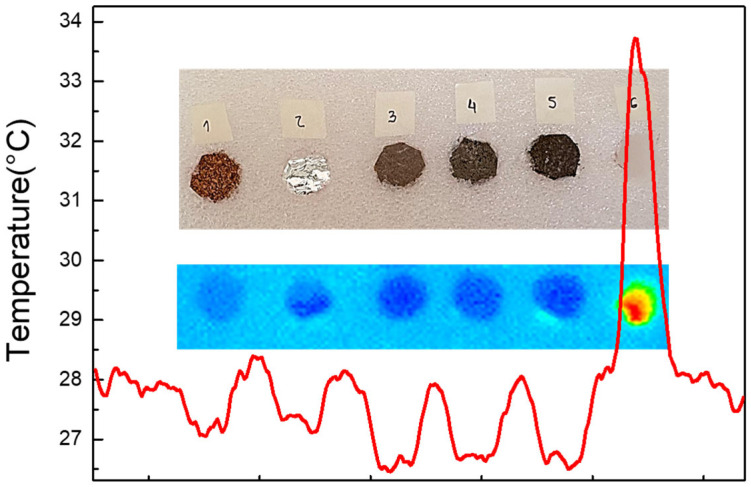
Surface temperature profiles of selected materials exposed to a hot plate under steady-state conditions: (1) cork, (2) polyethylene foam, (3) F-BP60, (4) F-BP60-25-HC, (5) F-BP60-25-CC, and (6) control.

To visualize the performance of thermal insulators, Figure 13 shows infrared thermographic images of the surfaces of different materials after thermal stabilization on a hot plate. The results reveal differences in steady-state surface temperature among the evaluated materials. The control sample (not insulated material) exhibits the highest temperature (33.71 °C), indicating minimal thermal resistance between the heat source and the exposed surface. In contrast, cork and polyethylene foam show significantly lower temperatures (28.20 °C and 28.38 °C, respectively), consistent with their well-known insulating performance. The F-BP60 sample reaches 28.16 °C, demonstrating effective thermal attenuation comparable to conventional materials. Differences between processing routes are observed for the biochar-containing composites. F-BP60-25-HC presents a surface temperature of 27.96 °C, while the cold-pressed counterpart reaches 28.03 °C. Although the temperature difference is modest, the hot-pressed sample exhibits a more uniform thermal profile, suggesting improved structural continuity and more homogeneous heat flux distribution. Overall, the thermographic results corroborate the thermal conductivity measurements, confirming that the inverse-vulcanized sulfur–soybean oil composites provide competitive insulation performance.

To further contextualize the thermal performance of the developed materials, a comparative summary of thermal conductivities for conventional and bio-based insulating systems is presented in Table 4. The best-performing biopolymer composites in this study exhibit thermal conductivities comparable to commercial insulators such as cork (≈0.039 W/(m·K) [72]) and polyethylene foam (≈0.035 W/(m·K) [73]). The FBP-60-C25 sample, in particular, achieved a λ_t_ of 0.0393 W/(m·K), placing it within the range of materials commonly used for thermal insulation, with the advantage that the material is fully obtained from renewable sources. Notably, even the cold-pressed variant, which exhibits slightly lower thermal conductivity due to its higher porosity, suffers from insufficient mechanical robustness, highlighting the relevance of processing-induced structure–property trade-offs. Moreover, compared to conventional polymeric insulators like polyester (0.050 W/(m·K) [74]), it approaches the thermal conductivity of air (0.024 W/(m·K) [75]), one of the most efficient insulating media. Compared with other bio-sourced polymer systems reported in the literature, the present materials exhibit significantly lower thermal conductivities. For instance, polylactic acid (PLA) films have been reported to display λ values in the range of 0.12–0.19 W/(m·K), while cellulose palmitate (CP) films show even higher values between 0.22 and 0.30 W/(m·K), depending on film thickness and morphology [76]. Porous foams with high sulfur content (50–90 wt%) and controlled porosity, produced by inverse vulcanization combined with template removal techniques, achieved values of ~0.032 W/(m·K), of the order reported in this work [35]. Notably, while that study relies on divinylbenzene (DVB) as cross-linker, the present system employs soybean oil, providing a renewable alternative. In addition to sulfur-based systems, the use of porous lignocellulosic and bio-derivative materials, such as cellulose foams, chitosan-based aerogels, and mycelium composites, has been widely reported, with thermal conductivities typically ranging from 0.03 to 0.06 W/(m·K), positioning them as sustainable alternatives to conventional polymeric insulators [77,78,79]. In this context, the inverse-vulcanized soybean oil and sulfur composites presented here demonstrate thermal performance that is fully competitive with state-of-the-art bio-insulation materials, while also offering additional advantages in terms of sulfur valorization and adjustable grid architecture and in the use of renewable feedstocks.

## 3. Materials and Methods

### 3.1. Materials

Commercial soybean oil (Natura^®^, General Deheza, Córdoba, Argentina) was used as received without further purification. Elemental sulfur (Sigma–Aldrich, Buenos Aires, Argentina) was employed as the comonomer.

### 3.2. Methods

#### 3.2.1. Synthesis of Sulfur–Soybean Oil Biopolymers

**Scheme 1:**
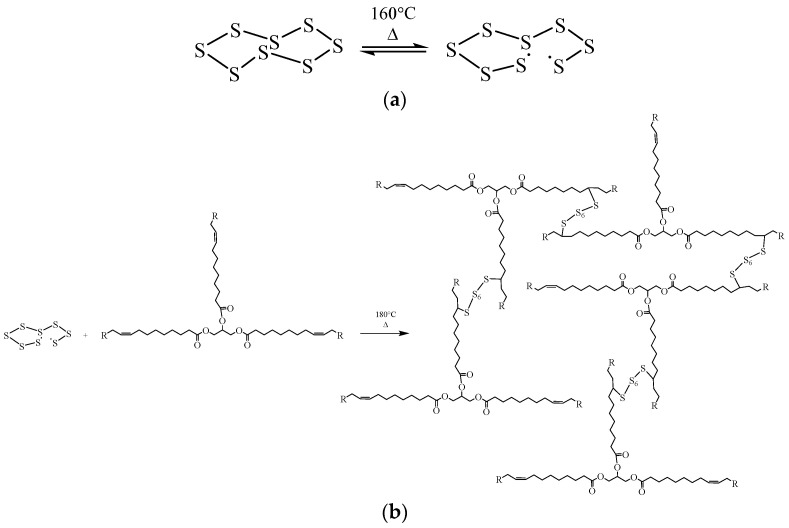
Inverse vulcanization mechanism: (**a**) ring-opening of elemental sulfur (S_8_) to form thyil radicals. (**b**) Cross-linking of soybean oil triglycerides via radical addition to C=C bonds. R represents generic alkyl chains.

The polymeric materials were synthesized by inverse vulcanization using elemental sulfur (S_8_) and commercial soybean oil (used without further purification) following the methodology previously reported by our group [2,3]. The reactions were carried out with a constant total mass of 30 g, varying the sulfur content from 20% to 80% by weight. In each case, an appropriate mass of sulfur was placed in a 250 mL glass beaker equipped with a magnetic stir bar and heated at 160 °C in a sand bath until it melted completely, forming a clear yellow liquid. A corresponding amount of pre-heated soybean oil (~160 °C) was then slowly added under continuous stirring. The reaction mixture was kept at 160 °C for 20 min. As the reaction progressed, the viscosity increased significantly and the texture of the material changed, indicating the formation of polymer. At this point, the stirring was stopped, and the vessel was removed from the heat. The resulting product was allowed to cool to room temperature, and a reddish-brown rubbery solid was obtained (Scheme 1). This procedure was repeated for formulations containing 20%, 30%, 40%, 50%, 60%, 70%, and 80% sulfur, and it produced materials named BP20, BP40, BP50, and BP60, respectively. All samples were stored in airtight containers at 4 °C in the dark before characterization.

#### 3.2.2. Biomass Filler Preparation

**Figure A1:**
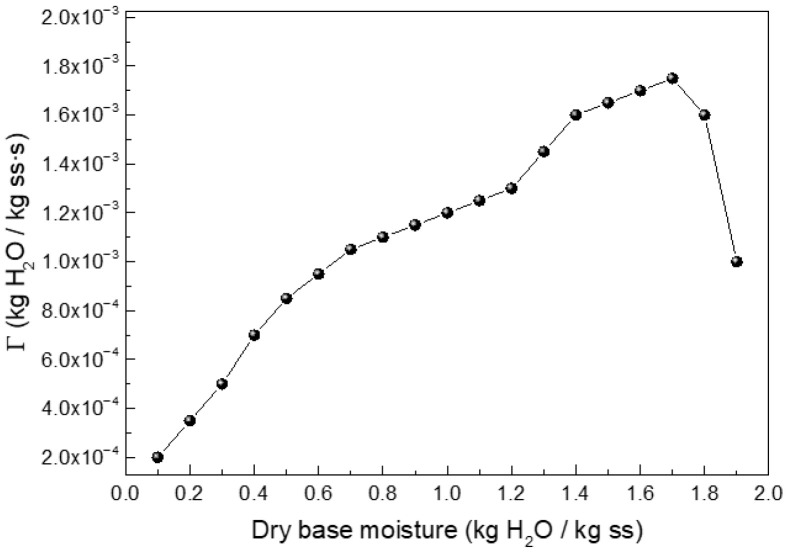
Drying rate barley curve.

Brewery waste, composed mainly of barley grains, and yeast from craft beer production were used as raw material to obtain the BC filler. The wet brewery waste was dried in a convection oven at 110 °C for 24 h until it reached a constant weight. The drying behavior was monitored by recording the mass loss at regular time intervals until stabilization. The drying curve (See Appendix A, Figure A1) was constructed to determine the kinetics and to establish the optimal pre-treatment conditions.

#### 3.2.3. Carbonization Process

Dried barley waste samples were placed in covered ceramic crucibles and subjected to carbonization in a muffle furnace under static air to obtain BC. Thermal treatment was performed at 200 °C for 2 h obtaining the BC named BC-200. After, the carbonized samples were weighed, and yield was calculated using Equation (2):

(2) $$Yield\left(\%\right)=\frac{m_{i}100}{m_{f}}$$

where $m_{i}$ is the initial dry mass and $m_{f}$ the final mass after carbonization. The resulting BC was ground using a blade mill and sieved, and the 180 μm fraction was selected for further use.

#### 3.2.4. Bio-Based Film Fabrication

Biopolymer films (F-BP) were prepared using a total mass of 15 g of biopolymer (BP) per formulation (BP20, BP40, BP50, and BP60) obtaining F-BP20, F-BP40, F-BP50, and FBP-60. Moreover, biocomposite films (F-BP-C) were obtained by incorporating BC particles into the biopolymer matrix at two different loadings, 15 and 25 wt%, producing films denoted as F-, F-BP40-C15, F-BP40-C25, F-BP60-C15, and F-BP60-C25. BC was mechanically ground and subsequently sieved to obtain particles with an average size of 180 µm.

Briefly, ground BP and a mixture of ground BP and BC were placed between two stainless-steel plates in a screw press and compacted at 17.35 MPa. The samples were then brought under reactive compression in an oven at 110 °C for 60 min [8]. Additionally, F-BP60-C25 formulation was prepared using two different processing routes—hot pressing at 110 °C (F-BP60-C25-HC) for 1 h and cold pressing at room temperature (F-BP60-C25-CC) for 7 days—using 15 g of BC and yielding uniform plates with comparable thicknesses (3.0 ± 0.1) mm.

**Scheme 2:**
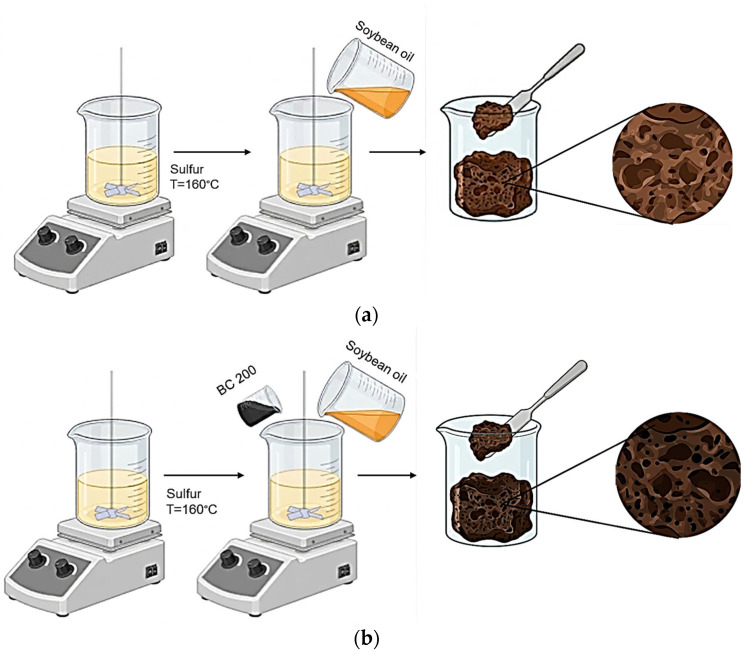
Pictorial representation of the synthesis of (**a**) BP and (**b**) F-BP-C under different processing conditions.

A comprehensive schematic representation of the overall fabrication process, including BP synthesis and biochar incorporation under different processing conditions, is presented in Scheme 2.

### 3.3. Materials’ Characterization

The chemical composition of the materials was analyzed by FTIR-ATR. The spectra were obtained by an ATR-FTIR Bruker Tensor 27 spectrometer (Bruker, Ettlingen, Germany) in absorbance mode. In order to obtain the spectra, the samples were dried for 48 h at 30 °C under dynamic vacuum. Duplicate spectra per sample were obtained with 20 scans per spectrum at a spectral resolution of 4 cm^−1^ in the wavenumber range from 4000 to 500 cm^−1^. As a reference, the background spectrum of air was collected before the acquisition of each sample spectrum.

Also, a Carl Zeiss EVO MA10 scanning electron microscope (Carl Zeiss, Oberkochen, Germany) with an integrated EDX system was used to examine the morphology of the materials. The samples were sputter-coated with a thin layer of gold for 90 s at a current of 40 mA and a pressure of 0.4 mbar under an argon atmosphere. The polymer morphology was examined through scanning electron microscopy. A voltage of 3 kV was applied for capturing scanning electron microscopy (SEM) images of the samples.

The thermo-responsive performance of materials was characterized via DSC. A Netzsch DSC-204-F1-Phoenix differential scanning calorimeter (Netzsch, Selb, Germany) equipped with a cooling device was used to perform the test. The samples were characterized at a 5.0 °C/min heating and cooling rate for 2 cycles from −10 °C to 140 °C to study the effect of material composition on thermal properties.

Static contact angle (CA) was measured using the optical tensiometer Theta Flow (Biolin Scientific, Gothenburg, Sweden). A droplet of bi-distilled water (5 μL) was placed on the surface of the film, and the CA experiments were measured three times for each sample. The analysis of the contact angle was performed using the One Attension^®^ software (version 4.0, Biolin Scientific); the informed value is the average of the 5 photographs.

**Scheme 3:**
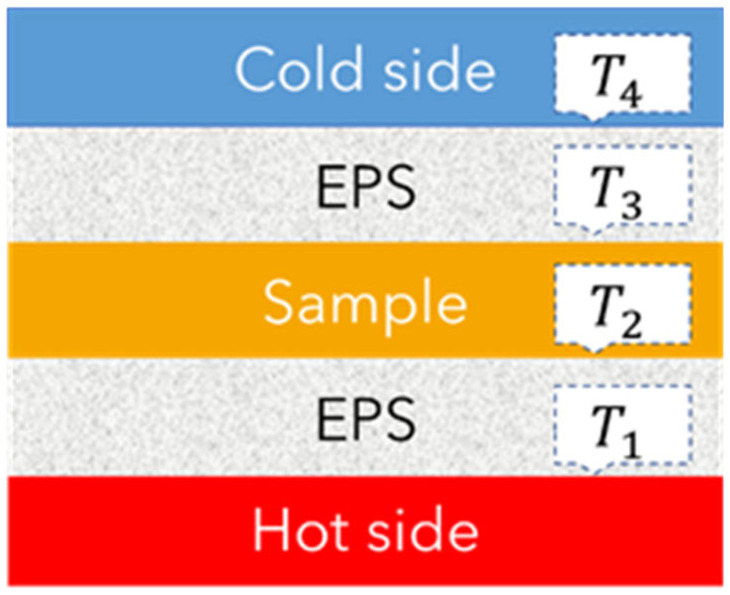
Thermocouple arrangement and layer structure in the heat flow measurement system.

The thermal conductivity (λ_t_) of the F-BP and F-BP-C was determined by the method described by Vitale et al. [80] Briefly, a modified comparative technique was employed based on ASTM standards (C177, C518, and E1225) [81,82]. The experimental setup consisted of a single-sided guarded hot plate set at 40 °C as the heat source and a cold source maintained at 20 °C using a circulating water reservoir. The sample was placed between reference plates made of expanded polystyrene (EPS, known thermal conductivity) and placed in an insulated box. The temperature at each interface was recorded using thermocouples strategically placed on the surfaces of the plates and the sample, as shown in Scheme 3. Temperature was monitored using Type K thermocouples (RS Components, Buenos Aires, Argentina) with an accuracy of ±0.1 °C.

Assuming one-dimensional heat flow in a steady state and negligible convective losses, the thermal conductivity of the sample was calculated using Fourier’s law [83]. Two estimates are calculated, one assuming heat flow from the hot side (Equation (3)) and the other from the cold side (Equation (4)):

(3) $$\lambda_{2hot}=\lambda_{1}A\frac{T_{1}-T_{2}}{T_{2}-T_{3}}\frac{L_{2}}{L_{1}}$$

(4) $$\lambda_{2cold}=\lambda_{3}A\frac{T_{3}-T_{4}}{T_{2}-T_{3}}\frac{L_{2}}{L_{3}}$$

The effective thermal conductivity of the intermediate layer is then obtained as the average of these two values (Equation (5)):

(5) $$\lambda_{t}=\frac{\lambda_{2hot}+\lambda_{2cold}}{2}$$

All samples were measured in triplicate, and the reported $\lambda_{t}$ values are expressed as mean ± standard deviation (*n* = 3), calculated from the values obtained on the hot and cold sides of each sample.

Tensile tests were conducted in accordance with the ASTM D638-10 standard, using Type V specimens [82]. The samples were precisely cut from each molded F-BP and F-BP-C using a precision cutter. The tests were carried out on a Mecmesin MultiTest 1-i universal testing machine (Mecmesin, Slinfold, UK) equipped with a 10 N load cell at a constant crosshead speed of 1 mm/min. Each measurement was performed in triplicate, and the tensile properties are reported as mean ± standard deviation (*n* = 3). Similarly, although no formal statistical tests were applied, the differences between samples were reproducible and exceeded the experimental variability.

Bulk density was determined by cutting rectangular F-BP or F-BP-C samples from each formulation into cubic specimens (1 cm^3^) using a precision blade. The dimensions of each specimen were measured with a digital caliper (±0.001 cm), and their mass was determined using an analytical balance (±0.0001 g). The density (ρ) was calculated by dividing the sample mass by its volume. Each sample was measured in triplicate, and the reported values correspond to the average.

Porosity (φ) was determined via water immersion under vacuum [2]. Dried materials were first weighed ($m_{1}$), and then the sample was fully submerged in deionized water within a sealed vacuum chamber for 10 min to ensure pore saturation at controlled temperature (25 °C). After vacuum treatment, the samples were wiped to remove surface water and reweighed ($m_{2}$). The apparent porosity (%) was estimated using Equation (6) [83,84,85]:

(6) $$Porosity\left(\%\right)=\left(\frac{m_{2}-m_{1}}{\rho_{water}V}\right)\times 100$$

where $\rho_{water}$ =1.00 (g/cm^3^), V is the specimen volume (cm^3^), and m_1_ and m_2_ are expressed in grams. All measurements were performed in triplicate, and the average values are reported.

Infrared thermal images were recorded using a Testo 868 thermographic camera (Testo SE & Co. KGaA, Titisee-Neustadt, Germany) with a thermal sensitivity of <0.08 °C at 30 °C, infrared resolution of 160 × 120 pixels, and a temperature range from −30 °C to 650 °C. Image analysis was performed using Testo IR Soft software (Version 4.3).

## 4. Conclusions

In this work, inverse-vulcanized soybean oil–sulfur composites reinforced with biochar were successfully developed and systematically evaluated as sustainable thermal insulation materials. Structural and thermal analyses confirmed the effective formation of a cross-linked sulfur–oil network, controlled sulfur incorporation, and the development of a heterogeneous microstructure governed by sulfur content, biochar loading, and processing route.

Microstructural organization and porosity were identified as the key parameters controlling thermal transport. The synergistic combination of air-filled voids, low-conductivity biochar domains, and multiscale interfacial phonon scattering enabled a significant suppression of heat transfer, yielding thermal conductivities as low as ~0.033–0.039 W/(m·K), values comparable to commercial insulating materials.

Although cold pressing promoted slightly lower thermal conductivity due to increased porosity, it compromised mechanical integrity. In contrast, hot pressing enhanced consolidation and interfacial cohesion, resulting in composites that achieve an optimal balance between mechanical robustness and thermal insulation performance.

Beyond their functional efficiency, these materials integrate renewable soybean oil, industrial sulfur surplus, and agro-industrial biochar residues within a scalable processing strategy, establishing a sustainable platform for lightweight bio-derived thermal insulation systems.

## Figures and Tables

**Table 1:** Determination of free sulfur by DSC.

F-BP	DSC Area (J/g)	Free S (%)
BP20	0.12	0.21
BP30	0.20	0.35
BP40	1.37	2.46
BP50	5.74	10.31
BP60	11.13	20.02
BP70	27.00	48.56
BP80	36.92	66.40
Pristine sulfur	55.60	100.00

**Table 2:** Thermal conductivity of sulfur–soybean oil F-BP and F-BP-C.

F-BP	λ_t_ [W/(m·K)]	F-BP-C	λ_t_ [W/(m·K)]
F-BP70	0.0404 ± 0.0013	F-BP60-C15	0.0463 ± 0.0017
F-BP60	0.0512 ± 0.0017	F-BP60-C25-HC	0.0393 ± 0.0012
F-BP50	0.0525 ± 0.0016	F-BP60-C25-CC	0.0333 ± 0.0010
F-BP40	0.0529 ± 0.0018	F-BP40-C15	0.0464 ± 0.0018
F-BP30	0.0610 ± 0.0021	F-BP40-C25	0.0473 ± 0.0019

Values are reported as mean ± standard deviation (*n* = 3).

**Table 3:** Summary of tensile properties of F-BP60-25 processed hot and cold.

Sample	Young Modulus (MPa)	Tensile Strength σ_max_ (MPa)	Elongation at Break (%)
F-BP60-25-CC	19.15 ± 1.25	0.088 ± 0.004	0.46 ± 0.05
F-BP60-25-HC	5.83 ± 0.73	0.185 ± 0.022	3.17 ± 0.02

Values are reported as mean ± standard deviation (*n* = 3).

**Table 4:** Thermal conductivity of conventional and bio-based insulating materials.

Material	Type/Structure	Thermal Conductivity (W/(m·K))	Reference
FBP-60-C25-CC	Sulfur–soybean oil composite	0.0393	This work
FBP-60-25-HC	Sulfur–soybean oil composite	~0.038–0.040	This work
Cork	Natural cellular solid	~0.039	[72]
Polyethylene foam	Synthetic polymer foam	~0.035	[73]
Polyester	Dense synthetic polymer	~0.050	[74]
Air	Gaseous phase	~0.024	[75]
PLA	Dense biopolymer	0.12–0.19	[76]
Cellulose derivatives	Dense biopolymer	0.22–0.30	[76]
Sulfur foams (DVB)	Porous sulfur polymer	~0.032	[35]
Cellulose foams	Porous lignocellulosic material	0.03–0.06	[77]
Mycelium composites	Bio-grown porous material	0.03–0.06	[78]
Chitosan aerogels	Biopolymer aerogel	0.03–0.05	[79]

## Data Availability

The data that support the findings of this study are available from the corresponding author upon reasonable request.

## Abbreviations

The following abbreviations are used in this manuscript:

BC	Biochar
F-BP	Sulfur–soybean oil biopolymer film
F-BP-C	Biochar-filled sulfur–soybean oil biocomposite film
DSC	Differential scanning calorimetry
SEM	Scanning electron microscopy
FT-IR	Fourier-transform infrared spectroscopy
wt%	Weight percentage
CA	Contact angle
λ_t_	Thermal conductivity
EPS	Expanded polystyrene
φ	Porosity
PLA	Polylactic acid
CP	Cellulose palmitate
DVB	Divinylbenzene
